# Network-based analysis identifies shared mechanisms between ischemic stroke and myocardial infarction and therapeutic ingredients of Buyang Huanwu Decoction

**DOI:** 10.3389/fgene.2026.1843679

**Published:** 2026-06-17

**Authors:** Shuaixin Zhang, Yue Lin, Wenke Xiao, Wei Chen, Sanyin Zhang

**Affiliations:** 1 School of Basic Medicine Sciences, Chengdu University of Traditional Chinese Medicine, Chengdu, China; 2 School of Pharmacy, Chengdu University of Traditional Chinese Medicine, Chengdu, China; 3 Innovative Institute of Chinese Medicine and Pharmacy, Chengdu University of Traditional Chinese Medicine, Chengdu, China; 4 Institute of Herbgenomics, Chengdu University of Traditional Chinese Medicine, Chengdu, China

**Keywords:** Buyang Huanwu Decoction, disease module, ischemic stroke, myocardial infarction, network proximity

## Abstract

**Introduction:**

Ischemic stroke (IS) and myocardial infarction (MI) share overlapping etiology and pathology, highlighting the need for dual-effective therapies. Buyang Huanwu Decoction (BHD) has shown efficacy against both diseases individually, but its core ingredients and dual therapeutic mechanisms remain unclear.

**Methods:**

Using network-based analysis, we identified shared targets and convergent pathways for IS and MI, prioritized BHD ingredients by network proximity, and performed functional enrichment analyses. Molecular docking validated interactions between the key target GSK-3β and core ingredients, and surface plasmon resonance (SPR) experimentally determined the binding affinity of the top-ranked ingredient, Myricanone, to GSK-3β.

**Results:**

Thirteen shared core targets were identified, with the “Lipid and atherosclerosis” pathway as the principal common mechanism. Ten key BHD ingredients with predicted dual therapeutic effects were prioritized. Among the shared targets, *GSK3B* emerged as a central node. Molecular docking showed stable binding of all core ingredients to GSK-3β, with Myricanone exhibiting the strongest affinity (−9 kcal/mol), which was confirmed by SPR (KD = 55.1 µM).

**Conclusion:**

These findings elucidate the shared mechanisms of BHD against IS and MI, supporting its potential as a multi-target phytotherapeutic strategy for ischemic cardio-cerebrovascular disorders.

## 1 Introduction

Ischemic stroke (IS) and myocardial infarction (MI) are major acute ischemic cardio-cerebrovascular diseases characterized by high morbidity and mortality, posing a substantial global health burden (Ajoolabady et al., 2021; Jernberg et al., 2015; Martin et al., 2025). These diseases share common risk factors and pathological bases, notably atherosclerosis (Björkegren and Lusis, 2022). Current standard therapies focus on timely reperfusion; however, their effectiveness is limited by a narrow therapeutic window (Scheldeman et al., 2024) and can lead to complications such as ischemia-reperfusion injury (Orellana-Urzúa et al., 2023) and bleeding risks (Zhao et al., 2022; Fernando et al., 2020). These limitations underscore the urgent need for complementary or alternative therapeutic strategies. In recent years, traditional Chinese medicine (TCM) has shown promising potential in alleviating symptoms and improving prognosis (Chen and Jin, 2023; Shao et al., 2022).

Buyang Huanwu Decoction (BHD), a classical formula, has demonstrated protective effects against cardio-cerebrovascular diseases via antioxidant (Liu et al., 2011; Cui et al., 2018), anti-apoptotic (Wang H. W. et al., 2011; Fan et al., 2006), and anti-inflammatory mechanisms (Wang W. R. et al., 2011; Dou et al., 2018). For instance, Huang et al. demonstrated that BHD alleviates IS-induced injury by inhibiting neuronal ferroptosis via activation of the Nrf2/GPX4 pathway (Huang et al., 2025). Zhu et al. reported that BHD ameliorates MI by promoting angiogenesis in the infarct border zone through the Caveolin-1/VEGF signaling pathway (Zhu J. Z. et al., 2019). Although these studies have confirmed the therapeutic efficacy of BHD for IS and MI individually, it remains unclear whether BHD exerts its effects against both diseases by modulating their shared core pathological mechanisms. Elucidating these shared mechanisms is essential for understanding the integrative pharmacology of BHD.

Although previous studies have attempted to explore the shared pathological basis of IS and MI, their findings remain limited by methodological constraints. For example, a prior bioinformatics analysis identified common differentially expressed genes (DEGs) and pathways between IS and MI (Wang et al., 2024). While this work provided initial insights into their potential shared mechanisms, the use of datasets containing post-treatment samples may have introduced therapeutic confounding, thereby obscuring genuine disease-related signals. To overcome this issue, the present study prioritized acute-phase, pre-treatment transcriptomic datasets to more accurately capture the intrinsic molecular features of both diseases.

**FIGURE 1:**
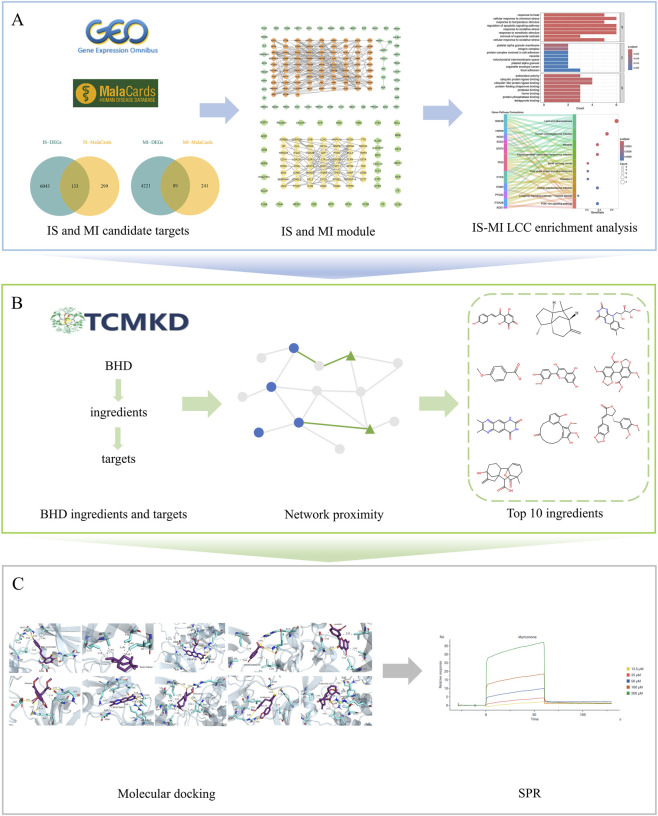
Overview of the network-based strategy for identifying shared mechanisms and core ingredients. **(A)** Identification of shared targets between IS and MI, followed by functional enrichment analysis to uncover their shared pathological mechanisms. **(B)** Screening of core ingredients from BHD against the shared targets using network proximity analysis. **(C)** Molecular docking of the key shared target with the core ingredients to validate binding affinity, and SPR validation of the top-ranked ingredient.

To systematically investigate the shared mechanisms of IS and MI and to elucidate the therapeutic potential of BHD, this study employed a network-based framework. First, disease modules were constructed on the human protein-protein interaction (PPI) network to identify shared core targets between IS and MI and to uncover the underlying common pathological pathways. Next, the network proximity between BHD targets and the shared disease modules was calculated to evaluate its potential efficacy against both conditions by using network proximity analysis (Guney et al., 2016; Cheng et al., 2019). Rheumatoid arthritis (RA) and its classical formula Guizhi Shaoyao Zhimu Decoction (GSZD) (Pan et al., 2025; Guo et al., 2016) were included as reference controls to enhance the specificity and interpretability of the analysis. Subsequently, the individual components of BHD were prioritized by assessing the network proximity of each ingredient to the shared core targets, highlighting the key bioactive ingredients. Finally, molecular docking was employed to examine the potential interactions and binding affinities of the core ingredients with critical target proteins. The top prediction was then validated experimentally using surface plasmon resonance (SPR). The overall study workflow is summarized in Figure 1.

## 2 Materials and methods

### 2.1 Data collection and processing

#### 2.1.1 Acquisition of disease transcriptomic data and disease-related genes

The standardized Medical Subject Heading terms “Ischemic Stroke”, “Myocardial Infarction”, and “Arthritis, Rheumatoid” were obtained from the Medical Subject Heading database (https://www.ncbi.nlm.nih.gov/mesh/). GSE datasets for IS and MI restricted to “*Homo sapiens*” were retrieved from the Gene Expression Omnibus (GEO) database (Edgar et al., 2002) using the keywords “Ischemic Stroke” and “Myocardial Infarction”, covering all entries up to 1 March 2025. One GEO dataset for RA was additionally collected using the keyword “Arthritis, Rheumatoid” and used as a control case.

The selection of GSE datasets adhered to strict inclusion criteria. All considered datasets were required to have clear citation sources and comprise either RNA-seq or microarray data. Each dataset should contain well-defined disease samples along with matched normal controls. Crucially, samples were collected during the acute phase of the disease. For IS, samples were collected within 24 h post-onset that corresponds to both the radiological acute phase (Birenbaum et al., 2011) and the extended treatment window for mechanical thrombectomy (Walter, 2022). For MI, samples were collected within 12 h post-onset, which follows the standard reperfusion window for STEMI (Scheldeman et al., 2024). To minimize the potential confounding effects of therapeutic interventions on transcriptomic profiles, samples without documented disease-modifying therapy were preferentially included. For RA, samples were collected from newly diagnosed RA patients prior to the initiation of disease-modifying antirheumatic drug therapy. Furthermore, only blood-derived mRNA sequencing data were included. Sample quality control was rigorously performed by employing hierarchical clustering based on Euclidean distance to identify and remove outliers. A minimum of three biological replicates per group after this filtering step was mandatory for subsequent analysis.

Based on these criteria, after quality control and selection of acute-phase samples, we preferentially included samples without documented disease-modifying therapy or those collected before any revascularization procedure. Accordingly, we obtained one IS dataset (GSE122709 (RNA-seq) (Zhu W. et al., 2019) with 5 disease samples and 5 control samples), three MI datasets (GSE109048 (microarray) (Gobbi et al., 2019) with 14 disease samples and 4 control samples, GSE60993 (microarray) (Park et al., 2015) with 13 disease samples and 7 control samples, and GSE61144 (microarray) (Park et al., 2015) with 7 disease samples and 10 control samples). In addition, one RA dataset (GSE274598 (RNA-seq) (Fresneda Alarcon et al., 2025) with 8 disease samples and 3 control samples) was processed following the same quality control pipeline and used to test the specificity of the network proximity approach. The details of the samples are listed in Supplementary Table S1. To ensure data quality and verify disease-control separation after preprocessing, we performed principal component analysis for each dataset using the prcomp () function in R (version 4.4.0). Results were visualized with confidence ellipses using the ggplot2 (version 3.5.2) (Wickham, 2016) and factoextra (version 1.0.7) packages (Supplementary Figure S1).

For data processing, all analyses were uniformly conducted using R (version 4.4.0). After removing outlier samples identified by hierarchical clustering, differential expression analysis was performed separately for each GSE dataset. RNA-seq count data were analyzed using the DESeq2 package (version 1.44.0) (Love et al., 2014), and microarray data were analyzed using the limma package (version 3.60.6) (Ritchie et al., 2015), with DEGs defined by an adjusted *p*-value < 0.05. mRNA annotations were based on the Human GRCh38.p13 reference genome. To integrate the DEG results across the three MI datasets, we took the union of all upregulated DEGs from the three datasets and the union of all downregulated DEGs from the three datasets, then removed any gene that appeared in both unions. The remaining genes, which were consistently either only upregulated or only downregulated, constituted the final MI DEG set. For IS and RA (each a single dataset), all DEGs were retained.

To further filter the DEGs and reduce the risk of false positives, disease-related genes for IS, MI, and RA were retrieved from the MalaCards database (https://www.malacards.org/, version 5.24) using the standardized disease terms “Stroke, Ischemic”, “Myocardial Infarction”, and “Rheumatoid Arthritis”. The DEGs for IS, MI, and RA were then intersected with their respective disease-related genes using the VennDiagram package (version 1.7.3) (Chen and Boutros, 2011) in R 4.4.0 to obtain candidate targets for each disease.

#### 2.1.2 Collection of herbal ingredients and targets

Using the TCMKD database (Xiao et al., 2025), we retrieved the ingredients and their corresponding targets for BHD (*Astragali Radix*, *Angelicae Sinensis Radix*, *Paeoniae Radix Rubra*, *Pheretima*, *Chuanxiong Rhizoma*, *Carthami Flos*, and *Persicae Semen*) as well as for the reference formula GSZD (*Cinnamomi Ramulus*, *Paeoniae Radix Alba*, *Anemarrhenae Rhizoma*, *Ephedrae Herba*, *Zingiberis Rhizoma Recens*, *Atractylodis Macrocephalae Rhizoma*, *Saposhnikoviae Radix*, *Aconiti Lateralis Radix Praeparata*, and *Glycyrrhizae Radix et Rhizoma*). A total of 1,391 ingredients were identified for BHD. Target information was available for 842 of these ingredients, and the union of their predicted or reported protein targets resulted in 8,756 unique targets (Supplementary Table S2A,B). For the reference formula GSZD, 9,805 unique targets were collected from the same database (Supplementary Table S2C).

### 2.2 Construction of disease-specific modules

The disease module proposed by Menche et al., suggests that proteins associated with a specific disease have a strong tendency to interact with one another, forming one or several interconnected subnetworks within the PPI network (Menche et al., 2015). In this study, we used the high-quality, integrated human PPI network constructed by Gysi et al. (Morselli Gysi et al., 2021) as the background network, which contains 18,505 nodes and 327,924 edges. Candidate disease targets were mapped onto the PPI network using Python (version 2.7.18). The largest directly interconnected subnetwork formed by these candidate targets was defined as the largest connected component (LCC). To determine whether the observed clustering of disease targets was greater than expected by chance, we generated 1,000 random target sets of equal size and computed the distribution of their LCC. The statistical significance of the observed LCC was assessed by calculating a *Z*-score according to Equation 1:

$$Z_{score}=\frac{{LCC}_{observed}-{\langle LCC}_{rand}\rangle }{\sigma \left({LCC}_{rand}\right)}$$ (1)

A *Z*-score > 1.6 indicates that the disease targets exhibit significantly stronger clustering than expected from random sets (Gan et al., 2023), thereby confirming the formation of a disease-specific module within the PPI network.

Gene symbols of IS, MI, and RA candidate targets were translated to Entrez IDs using the org.Hs.eg.db package (version 3.19.1). Disease modules for IS, MI, and RA were constructed separately, and their corresponding LCC targets were identified. The disease modules were visualized using Cytoscape 3.10.3. The intersection between the IS LCC and MI LCC was defined as the shared IS-MI LCC target set.

### 2.3 Network proximity calculation

Network proximity quantifies the relationship between drug targets and disease-associated proteins within the PPI network by measuring the average shortest distance from each drug target to its closest disease protein (Guney et al., 2016). Using the integrated human PPI network from Gysi et al. (Morselli Gysi et al., 2021) as the background interactome, we mapped the targets of disease and Chinese medicine or ingredients onto the network using Python (version 2.7.18). The network proximity was quantified using the closest distance metric *d*_*c*_. Let *A* represent the set of disease targets and *B* the set of Chinese medicine or ingredient targets. For any disease target *a∈A* and ingredient target *b∈B*, *d*(*a, b*) represents the shortest path length between them in the PPI network. The average minimal distance from set B to set A was calculated as Equation 2:

$$d_{c}\left(A,B\right)=\frac{1}{\left\|B\right\|}\sum_{b\in B}\min_{a\in A}d\left(a,b\right)$$ (2)

To assess the statistical significance of the observed proximity, we generated 1,000 random pairs of target sets matched to A and B in both size and degree distribution. This was achieved using a degree-binning node resampling method. All nodes in the network were first grouped into bins according to their degree, each bin containing at least 100 nodes. For each original target set (A or B), we determined how many nodes fell into each degree bin. Then, for each of the 1,000 iterations, we randomly sampled (without replacement) the same number of nodes from each corresponding bin to form a randomized set. This procedure ensures that the randomized control sets preserve the degree distribution of the original sets. The shortest-path distance was then computed for each random pair. A *Z*-score, *Z*_*dc*_, was calculated as Equation 3:

$$Z_{dc}=\frac{d_{c}-\mu_{dc}\left(A,B\right)}{\sigma_{dc}\left(A,B\right)}$$ (3)

where *μ*_*dc*_ and *σ*_*dc*_ represent the mean and standard deviation of the distances from the random pairs. A *Z*_*dc*_ < 0 indicates that the observed distance is significantly smaller than expected by chance (Gan et al., 2023), suggesting therapeutic potentials. A more negative *Z*_*dc*_ value reflects closer drug-disease relationships and stronger predicted efficacy.

By converting gene symbols of BHD and GSZD targets to Entrez IDs with the org.Hs.eg.db package (version 3.19.1), we calculated the network proximity for three target-set pairs: BHD targets and IS-MI LCC targets, BHD targets and RA LCC targets, as well as GSZD targets and IS-MI LCC targets, respectively. The resulting *Z*_*dc*_ values were compared to evaluate the potential therapeutic advantages of BHD for IS and MI.

We further calculated the network proximity between each individual ingredient’s targets in BHD and the IS-MI LCC targets. Ingredients were ranked in ascending order of their *Z*_*dc*_ values, and the top 10 ingredients with the closest proximity were designated as the core active ingredients of BHD against IS and MI.

### 2.4 Functional enrichment analysis

Gene Ontology (GO) terms and Kyoto Encyclopedia of Genes and Genomes (KEGG) pathway enrichment analyses were performed using the clusterProfiler package (version 4.14.6) (Yu et al., 2012) in R (version 4.4.0). The results were visualized with ggplot2 (version 3.5.2) (Wickham, 2016). To identify the primary shared mechanism, we performed functional enrichment on the IS-MI LCC targets (adjusted *p*-value < 0.05) and designated the top-ranking KEGG pathway as the target pathway. Subsequently, the same analysis was applied to the core ingredient targets. KEGG pathways shared between the IS-MI LCC targets and the core ingredient targets were considered the therapeutic pathways through which BHD may act on both diseases. The intersection of disease targets and ingredient targets enriched in the shared pathway were defined as the key targets.

### 2.5 Molecular docking validation

The interaction strength between the core ingredients and key targets was evaluated using molecular docking. Proteins were selected based on the key targets’ gene names from the UniProt database (UniProt Consortium, 2025), with filtering limited to reviewed (Swiss-Prot) entries from *Homo sapiens*. Their three-dimensional structures were retrieved from the RCSB Protein Data Bank database (Burley et al., 2023), with a preference for those determined by X-ray crystallography and with higher resolution. Proteins were pre-processed in PyMOL (version 3.1.5.1), and the binding pocket was defined according to the location of the co-crystallized ligand. For the core ingredients, their structure data files were obtained from the PubChem database (Kim et al., 2025) using PubChem CID numbers from TCMKD. When three-dimensional conformers were unavailable, two-dimensional structures were converted to three-dimensional. All ligand structure data files were converted to MOL2 format using Open Babel (version 3.1.1) (O'Boyle et al., 2011). Both ligands and proteins were then processed using AutoDockTools (version 1.5.7) (Forli et al., 2016) and stored in PDBQT format. AutoDock Vina (version 1.2.7) was employed to conduct batch molecular docking. AutoDock Vina generated up to 20 binding poses per docking run and reported the root-mean-square deviation (RMSD) of each pose relative to the best-scoring pose. A binding energy (BE) < −5.0 kcal/mol was used as the threshold to indicate a strong and reliable ligand-receptor interaction (Wang et al., 2025). Protein-Ligand Interaction Profiler (Schake et al., 2025) was utilized to analyze the molecular docking results for protein-ligand interactions such as hydrogen bonds, hydrophobic interactions, and salt bridges, which were then visualized in PyMOL.

### 2.6 SPR assay

To validate the direct interaction between the top-ranked core ingredient Myricanone and the key target GSK-3β, SPR was employed to measure their binding affinity. SPR assays were carried out on a Biacore 1K system (Cytiva) using a CM5 sensor chip (Cytiva). The core target protein GSK-3β (10044-H07B, Sino Biological) and the core ingredient Myricanone (B30409, Shanghai Yuanye) were purchased from commercial sources.

To determine the optimal pH for immobilization, GSK-3β was diluted to 32.4 μg/mL in 10 mM sodium acetate buffers at pH 5.0 and 4.5, respectively. The pre-concentration experiment identified pH 4.5 as the optimal condition. Subsequently, the protein was immobilized on the chip surface at the optimal pH via amine coupling. The running buffer was prepared by diluting 10× HBS-EP + buffer (BR100669, Cytiva) to 1× and then mixing with DMSO (D8371, Solarbio) to achieve a final DMSO concentration of 5%. Solvent correction was performed using buffers containing 4.5% and 5.8% DMSO to account for the 5% DMSO in running buffer. The sample was prepared as follows: A 10 mM stock solution of Myricanone was first prepared in DMSO. A 500 µM intermediate solution was prepared by diluting the stock solution 20-fold with 1× HBS-EP + buffer. Different concentrations of the analyte were then obtained by diluting the intermediate with running buffer to final concentrations of 200, 100, 50, 25, and 12.5 µM. The binding assay was performed once, with an association time of 60 s, a dissociation time of 60 s, and a flow rate of 30 μL/min. The equilibrium dissociation constant (KD) was obtained by globally fitting the sensorgram data to a 1:1 Langmuir binding model using Biacore Insight Evaluation Software.

## 3 Results

### 3.1 Identification of candidate targets for IS and MI

**FIGURE 2:**
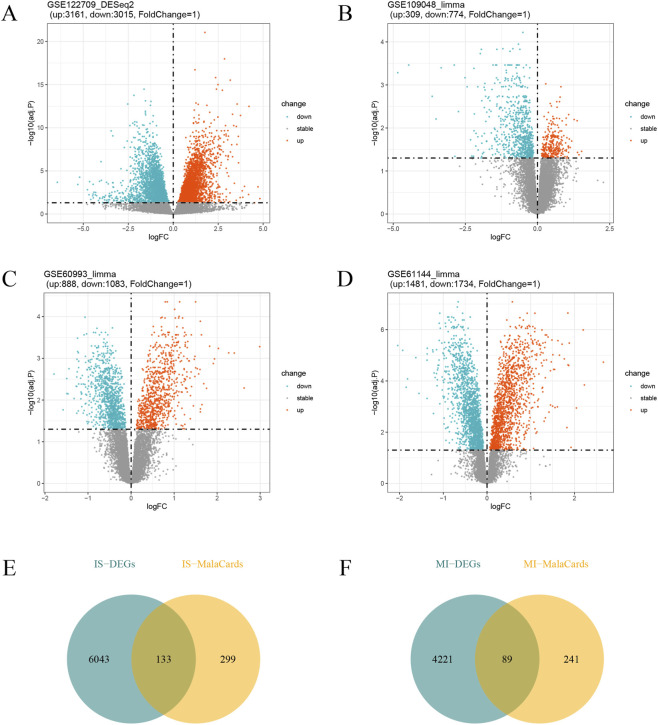
Candidate targets for IS and MI. **(A–D)** Volcano plots of DEGs (adjusted *p*-value < 0.05) in IS dataset GSE122709, MI datasets GSE109048, GSE60993, and GSE61144, respectively. **(E, F)** Intersection analyses between DEGs and disease-related genes for IS and MI, respectively.

To establish a high-confidence set of disease-relevant targets for subsequent network analysis, we first identified candidate targets for IS, MI, and the reference disease RA through a multi-step filtering process. Differential gene analysis of the IS dataset (GSE122709) obtained 6,176 DEGs (3,161 up-regulated, 3,015 down-regulated; Figure 2A). For MI, 1,083 DEGs were identified in GSE109048 (309 up-regulated, 774 down-regulated; Figure 2B); 1,971 DEGs were identified in GSE60993 (888 up-regulated, 1,083 down-regulated; Figure 2C); and 3,215 DEGs were identified in GSE61144 (1,481 up-regulated, 1,734 down-regulated; Figure 2D). Integration of the three MI datasets yielded a final set of 4,310 MI DEGs (1,862 up-regulated, 2,448 down-regulated). Analysis of the RA dataset (GSE274598) identified 2,484 DEGs (1,045 up-regulated, 1,439 down-regulated) (all adjusted *p*-value < 0.05; Supplementary Table S3A). From the MalaCards database, we obtained 432, 330, and 324 disease-related genes for IS, MI, and RA, respectively (Supplementary Table S3B). Intersection analysis of the respective DEGs with disease-related genes yielded 133, 89, and 74 candidate targets for IS, MI, and RA (Supplementary Table S3C). The overlaps for IS and MI are shown in the Venn diagrams in Figures 2E,F, respectively.

### 3.2 Construction of disease modules reveals shared core targets in IS and MI

**FIGURE 3:**
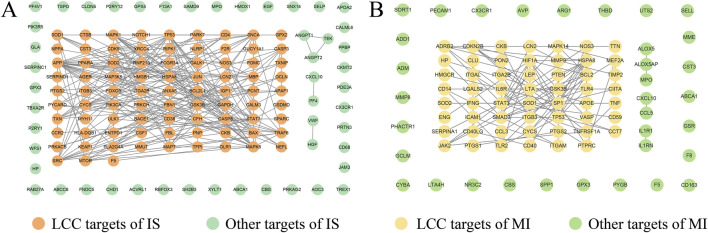
Disease modules for IS and MI. **(A)** IS disease module. Orange nodes represent LCC targets. Green nodes represent targets with *d*_*s*_ = 1 that are not included in the LCC, whereas green nodes in the outermost layer represent targets with *d*_*s*_ ≥ 2 to other targets. Gray edges indicate targets and targets interactions. **(B)** MI disease module. Yellow nodes represent LCC targets. Cyan nodes represent targets with *d*_*s*_ = 1 that are not included in the LCC, whereas cyan nodes in the outermost layer represent targets with *d*_*s*_ ≥ 2 to other targets. Gray edges indicate targets and targets interactions.

We next constructed disease modules from the candidate targets to identify core targets underlying each disease. To ensure that subsequent assessments of BHD’s therapeutic potential were attributable to the specific pathophysiological context of IS and MI rather than to methodological artifacts, a parallel analysis was performed for the reference disease, RA, to construct a comparable disease module under identical computational parameters. The gene symbols of all the IS, MI, and RA candidate targets were converted to Entrez IDs. The 133 candidate IS targets were mapped onto the PPI network to construct the IS module. As shown in Figure 3A, the IS module contained a largest LCC of 84 interconnected targets (*Z* = 27.39, *P* < 0.0001; Supplementary Table S4A). Among the 89 MI candidate targets, 88 were localized in the PPI network, forming the MI module. The MI module (Figure 3B) comprised an LCC of 55 interconnected targets (*Z* = 37.61, *P* < 0.0001; Supplementary Table S4B). Similarly, localization of the 74 RA candidate targets in the PPI network produced an RA module containing an LCC of 53 targets (*Z* = 62.54, *P* < 0.0001; Supplementary Table S4C). The formation of these highly significant, cohesive subnetworks (all *P* < 0.0001) indicates that the candidate targets for each disease are not randomly distributed but form core functional units within the human interactome, thereby supporting their biological relevance to the respective pathophysiology.

Intersection analysis between the IS LCC targets and MI LCC targets identified 13 shared targets: *CKB*, *GSK3B*, *HSPA8*, *ITGA2B*, *ITGB3*, *LCN2*, *NOS3*, *PTGS2*, *SOD1*, *SOD2*, *STAT3*, *TP53*, and *CYCS*. These shared targets highlight a convergent molecular network architecture underlying the pathophysiology of both IS and MI, suggesting potential common mechanisms and cross-disease therapeutic opportunities.

### 3.3 Enrichment analysis of the IS-MI LCC targets reveals the lipid and atherosclerosis pathway as a key shared mechanism

**FIGURE 4:**
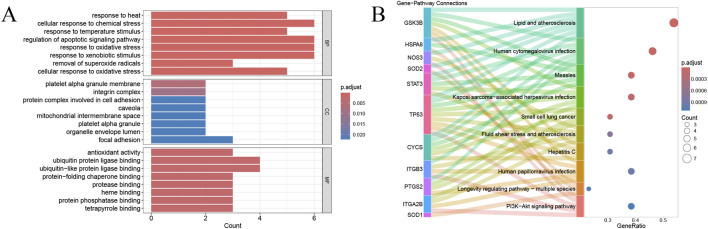
Enrichment analysis of the IS-MI LCC targets. **(A)** Top eight significantly enriched GO terms (BP, CC, MF) ranked by adjusted *p*-value. **(B)** Top ten significantly enriched KEGG pathways ranked by adjusted *p*-value.

To elucidate the biological mechanisms shared by IS and MI, we performed functional enrichment analysis on the 13 IS-MI LCC targets. The significantly enriched GO terms, namely Biological Process (BP), Cellular Component (CC), and Molecular Function (MF), were ranked by adjusted *p*-value. The top eight terms from each category are presented in Figure 4A, and all terms with adjusted *p*-value < 0.05 are listed in Supplementary Table S5A. In the GO enrichment analysis, BP were predominantly associated with oxidative stress response and apoptotic regulation, including “response to oxidative stress”, “removal of superoxide radicals”, and “regulation of apoptotic signaling pathway”. CC were enriched in structures involved in vascular adhesion, secretion, and organelle organization, such as “platelet alpha granule membrane”, “integrin complex”, “focal adhesion”, and “mitochondrial intermembrane space”. MF were enriched in antioxidant activities and protein modification binding, such as “antioxidant activity”, “ubiquitin protein ligase binding”, and “protease binding” (adjusted *p*-value < 0.05).

KEGG pathway analysis identified significant enrichment pathways, including “Lipid and atherosclerosis”, “PI3K-Akt signaling pathway”, and “Fluid shear stress and atherosclerosis” (adjusted *p*-value < 0.05, Figure 4B; Supplementary Table S5B). Among these, the “Lipid and atherosclerosis” pathway was the most significantly enriched, with seven out of the 13 targets (*GSK3B*, *HSPA8*, *NOS3*, *SOD2*, *STAT3*, *TP53*, *CYCS*) mapped to this pathway. Together, these results highlight the “Lipid and atherosclerosis” pathway as the key shared mechanism underlying the pathophysiological convergence of IS and MI.

### 3.4 BHD demonstrates therapeutic potential for IS and MI compared to RA

To evaluate the specificity of BHD’s therapeutic potential for IS and MI, we performed a network proximity analysis using RA and its representative formula GSZD as comparative controls. The gene symbols of BHD targets were translated to 8,716 Entrez IDs, and the gene symbols of GSZD targets were translated to 9,768 Entrez IDs (Supplementary Table S6A).

The network proximity *Z*_*dc*_ value was −0.284 for BHD targets versus IS-MI LCC targets, 0.344 for BHD targets versus RA LCC targets, and −0.244 for GSZD targets versus IS-MI LCC targets (Supplementary Table S6B). Since a smaller *Z*_*dc*_ value represents a closer relative distance, this result implies that BHD showed the closest proximity to the IS-MI module. Collectively, these results indicate that, from a network topology perspective, BHD has a markedly stronger therapeutic association with IS and MI than with RA, and also exhibits greater specificity for IS-MI pathology than the reference formula GSZD.

### 3.5 Network proximity prioritizes core ingredients of BHD for the treatment of IS and MI

To identify the core ingredients of BHD with potential therapeutic effects against IS and MI, we calculated the network proximity of each ingredient’s targets to the IS-MI LCC targets. The gene symbols of BHD’s 842 ingredient targets were first converted to Entrez IDs (Supplementary Table S7A). Network proximity was then calculated individually for each of the 842 ingredients against the IS-MI LCC targets. After excluding one ingredient whose sole target was absent from the PPI network, 841 ingredients remained for analysis. A total of 841 network proximity results were obtained (Supplementary Table S7B), among which 570 exhibited *Z*_*dc*_ < 0. Based on the lowest *Z*_*dc*_ values, the top 10 ingredients were identified as the core ingredients. Their names, PubChem CID, together with the closest distance *d*_*c*_ and standardized network proximity *Z*_*dc*_ values, are detailed in Table 1. These ingredients represent the primary material basis for BHD’s therapeutic effects against both IS and MI, highlighting their potential contributions to the formula’s multi-target pharmacology.

**TABLE 1:** The top 10 ingredients of BHD characterized by the lowest *Z*_*dc*_ values.

Herbal ingredients	CID	*d*_*c*_	*Z*_*dc*_
3,5-dihydroxy-2-[(2E)-3-(4-hydroxyphenyl)prop-2-enoyl]cyclohexa-2,5-diene-1,4-dione	131833009	0.500	−4.923
beta-Cedrene	11106485	0.000	−4.533
7,8-dimethyl-10-((2R,3R,4S)-2,3,4,5-tetrahydroxypentyl)benzo [g]pteridine-2,4(3H,10H)-dione	6759	0.000	−4.533
4-Methoxybenzoate	3783514	0.800	−4.421
peonidin	441773	1.250	−4.352
Bifendate	108213	0.900	−4.346
7,8-dimethyl-1H-pyrazino [2,3-g]quinazoline-2,4-dione	21786815	1.000	−4.216
Myricanone	161748	1.130	−4.200
Suchilactone	132350840	1.167	−4.187
Gibberellin A95	312804	1.000	−4.179

### 3.6 Targets of the core ingredients converge on the shared lipid and atherosclerosis pathway

To explore whether BHD exerts its therapeutic effects against IS and MI by modulating their shared molecular mechanisms, we performed functional enrichment analysis on the 10 core ingredients’ targets. Collectively, these ingredients targeted 41 unique genes (Supplementary Table S8A). GO and KEGG enrichment analyses were conducted on these targets (adjusted *p*-value < 0.05, Supplementary Table S8B, C). In the GO enrichment analysis, the significantly enriched terms were ranked by adjusted *p*-value. The top eight terms (or all terms if fewer than eight) from each category are presented in Supplementary Figure S2A, and all terms with adjusted *p*-value < 0.05 are listed in Supplementary Table S8B. Specifically, BP were significantly associated with hormone signaling and nuclear receptor pathways, including “nuclear receptor-mediated signaling pathway”, “hormone-mediated signaling pathway”, and “peroxisome proliferator activated receptor signaling pathway”. CC terms were enriched in membrane channel complexes and endoplasmic reticulum structures, such as “voltage-gated sodium channel complex”, “monoatomic ion channel complex”, and “ER membrane insertion complex”. MF were terms highlighted receptor binding and transcriptional regulation, including “nuclear receptor activity”, “ligand-activated transcription factor activity”, and “nuclear steroid receptor activity” (Supplementary Figure S2A; Supplementary Table S8B). KEGG enrichment revealed significant overrepresentation in multiple pathways, including “Lipid and atherosclerosis”, “Calcium signaling pathway”, “Adrenergic signaling in cardiomyocytes”, and “Cellular senescence” (Supplementary Figure S2B; Supplementary Table S8C). Notably, the “Lipid and atherosclerosis” pathway overlapped with the previously identified shared pathway between IS and MI. Within this pathway, five core targets of BHD ingredients were enriched, namely *PPARG*, *GSK3B*, *CALM3*, *MAPK14*, and *RXRA*. This convergence of core ingredients’ targets on the key shared disease pathway supports the hypothesis that BHD treats both IS and MI primarily by modulating the “Lipid and atherosclerosis” pathway.

**FIGURE 5:**
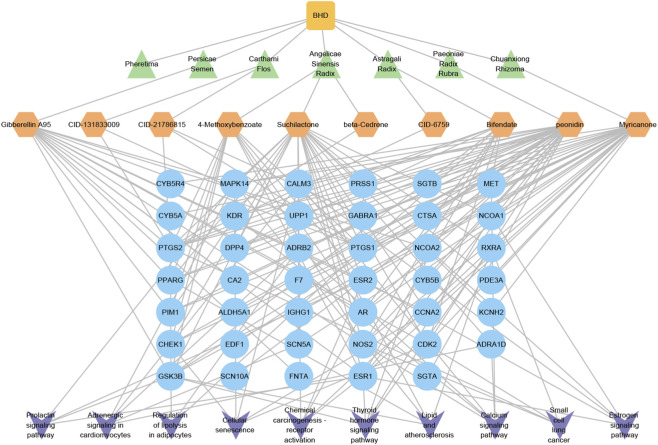
The BHD-herb-ingredient-target-pathway network. Square nodes (yellow) represent BHD; triangular nodes (green) represent herbs; hexagonal nodes (orange) represent ingredients; circular nodes (blue) represent targets; arrows (purple) represent pathways; lines (grey) indicate interactions between components.

To provide a holistic view of these interactions, we constructed an integrated network linking the BHD formula, its constituent herbs, the core ingredients, their targets, and the key pathway (Figure 5). This network visually captures the multi-ingredient, multi-target hierarchical framework of BHD and illustrates how the formula collectively converges on a central pathogenic mechanism, reinforcing the systems-level pharmacological rationale for its therapeutic efficacy.

### 3.7 Molecular docking validates stable binding of core ingredients to key targets in the lipid and atherosclerosis pathway

**FIGURE 6:**
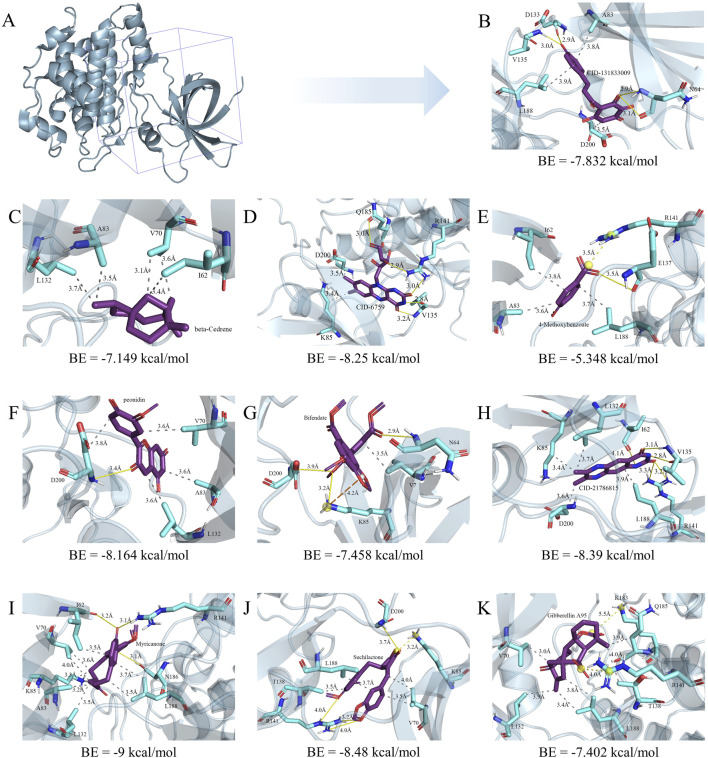
Molecular docking results between GSK-3β and 10 core ingredients. **(A)** Overview of GSK-3β binding pocket (blue). **(B–K)** Individual docking poses of CID-131833009, beta-Cedrene, CID-6759, 4-Methoxybenzoate, peonidin, Bifendate, CID-21786815, Myricanone, Suchilactone, and Gibberellin A95, respectively. Interactions are indicated as follows: yellow solid lines: hydrogen bonds; grey dashed lines: hydrophobic interactions; yellow dashed lines: salt bridges; orange dashed lines: π-cation interactions.

Among the shared targets, GSK-3β (PDB ID: 1J1B) was selected for molecular docking because it represents the intersection of the seven IS-MI LCC targets and five core ingredient targets within the “Lipid and atherosclerosis” pathway. Molecular docking was performed between GSK-3β and the 10 core ingredients. Figure 6A provides a detailed view of the binding pocket, while Figures 6B–K show the specific docking poses for each ingredient. Results of molecular docking showed good binding affinity of all 10 core ingredients to GSK-3β, with binding energies below −5 kcal/mol (Figure 6). The optimal binding energies and interacting residues for each ingredient are provided in Supplementary Table S9. The RMSD between the best-scoring pose and the second-best pose for the 10 core ingredients ranged from 0.06525 to 2.288 Å. Seven out of ten values were below 2.0 Å, indicating generally good convergence of the docking runs (Supplementary Table S9). In molecular docking, a more negative binding energy indicates stronger binding affinity. Notably, Myricanone demonstrated the strongest binding affinity to GSK-3β, with a binding energy of −9 kcal/mol, indicating stable and potentially biologically relevant interactions. These findings suggest that the core ingredients primarily act on GSK-3β, supporting the hypothesis that BHD exerts therapeutic effects against IS and MI by directly modulating GSK-3β within the shared “Lipid and atherosclerosis” pathway. Overall, the docking results provide molecular-level evidence for the network-based predictions, linking ingredient-target interactions to the convergent disease mechanism.

### 3.8 SPR assay validates the direct binding of myricanone to GSK-3β

**FIGURE 7:**
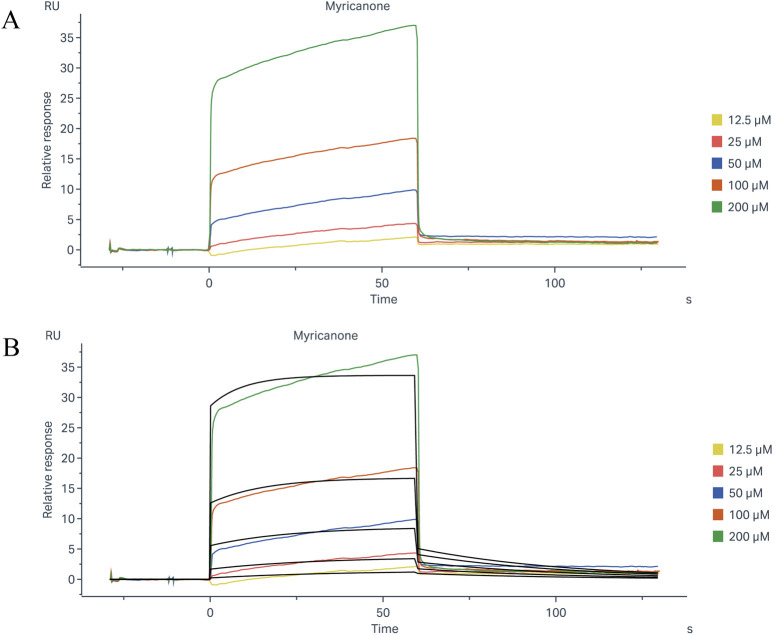
SPR analysis of Myricanone binding to GSK-3β. **(A)** Sensorgrams of Myricanone at various concentrations binding to immobilized GSK-3β. **(B)** Global fitting of the sensorgrams to a 1:1 Langmuir binding model. Lines in different colors correspond to concentration gradients, and black lines represent the fitted curves.

To experimentally validate the molecular docking prediction, we measured the binding affinity between the key shared target GSK-3β and Myricanone using SPR. Sensorgrams demonstrated clear concentration-dependent binding of Myricanone to the immobilized GSK-3β (Figure 7). A KD of 55.1 µM was obtained by fitting the sensorgram data to a 1:1 Langmuir interaction model. Detailed kinetic parameters are provided in Supplementary Table S10. This result provides direct experimental confirmation that Myricanone, a core ingredient of BHD, can interact directly with GSK-3β with measurable affinity, thereby offering crucial experimental support for the computational predictions obtained from network proximity analysis and molecular docking.

## 4 Discussion

IS and MI are severe cardio-cerebrovascular diseases sharing common risk factors and pathological features, including atherosclerosis and systemic inflammation (Björkegren and Lusis, 2022; DeLong et al., 2022; Ong et al., 2018). Although previous bioinformatics studies have suggested associations between IS and MI (Wang et al., 2024; Feng et al., 2022), the precise shared molecular mechanisms remain incompletely elucidated. In this study, we implemented an integrated network-based strategy to systematically identify the common pathogenic pathways between IS and MI, thereby deciphering the mechanistic basis of BHD in treating both conditions.

To minimize potential interference from reperfusion therapy and ensure the validity of downstream analysis, we selected IS and MI samples from the GEO database that were collected prior to treatment and within 24 and 12 h of symptom onset, respectively. Differential expression data were intersected with disease-related genes from MalaCards to construct IS and MI modules. Through network-based analysis, we identified 13 shared core targets between the two diseases (*CKB*, *GSK3B*, *HSPA8*, *ITGA2B*, *ITGB3*, *LCN2*, *NOS3*, *PTGS2*, *SOD1*, *SOD2*, *STAT3*, *TP53*, and *CYCS*). These targets span multiple pathological processes, including energy metabolism (*CKB*) (He et al., 2024), platelet aggregation and thrombosis (*ITGA2B*, *ITGB3*) (Ding et al., 2024), oxidative stress (*SOD1*, *SOD2*) (Baechler et al., 2019), inflammation (*STAT3* (Hillmer et al., 2016), *PTGS2* (Martín et al., 2023), *LCN2* (Li et al., 2023)), mitochondrial apoptosis (*TP53* (Voskarides and Giannopoulou, 2023), *CYCS* (Baechler et al., 2019)), endothelial dysfunction (*NOS3*) (Hwang et al., 2023), cell survival and signal transduction (*GSK3B*) (Sharma et al., 2021), and protein homeostasis and cytoprotection (*HSPA8*) (Alecki et al., 2024). The convergence of these targets on such pathological processes strongly supports their biological plausibility as shared molecular nodes underlying both IS and MI.

Functional enrichment analysis of the 13 shared core targets revealed that GO terms were associated with oxidative stress, apoptosis, and vascular adhesion, while KEGG pathways included “Lipid and atherosclerosis”, the “PI3K-Akt signaling pathway”, and the “Fluid shear stress and atherosclerosis”. The “Lipid and atherosclerosis” pathway was the most significantly enriched, encompassing *GSK3B*, *HSPA8*, *NOS3*, *SOD2*, *STAT3*, *TP53*, and *CYCS*. This finding is consistent with the established understanding that atherosclerosis serves as a common pathological basis for both diseases (Björkegren and Lusis, 2022). It is noteworthy that the “PI3K-Akt signaling pathway” (Zhao et al., 2021) and the “Fluid shear stress and atherosclerosis pathway” (Björkegren and Lusis, 2022) are well-established upstream regulatory pathways of lipid metabolism, inflammatory activation, and endothelial dysfunction. Their co-enrichment provides an upstream mechanistic explanation for our core finding of the shared “Lipid and atherosclerosis” pathway.

Network proximity analysis confirmed that BHD exhibits greater therapeutic potential for IS and MI compared to RA. Using this approach, we identified 10 core ingredients from BHD with potential anti-IS and anti-MI effects, including CID-131833009, beta-Cedrene, CID-6759, 4-Methoxybenzoate, peonidin, Bifendate, CID-21786815, Myricanone, Suchilactone and Gibberellin A95. To explore their mechanisms, we found that the 10 ingredients collectively targeted 41 genes, and five of them (*PPARG*, *GSK3B*, *CALM3*, *MAPK14*, *RXRA*) were significantly enriched in the shared “Lipid and atherosclerosis” pathway, suggesting a potentially synergistic effect of BHD through multi-ingredient, multi-target regulation. Notably, some of these ingredients have been implicated in cardio-cerebrovascular protection by previous studies. For example, Bifendate has been reported as a potential therapeutic ingredient for IS (Wang et al., 2021), and Myricanone may confer protection against MI by targeting *STAT3* (Yang et al., 2025).

Among these shared targets, *GSK3B* is the shared target between the seven IS-MI LCC targets and five core ingredient targets enriched in the “Lipid and atherosclerosis” pathway. *GSK3B* encodes a serine/threonine kinase that regulates glucose homeostasis and is implicated in energy metabolism, inflammation, ER stress, mitochondrial dysfunction, and apoptosis. Previous studies have shown that *GSK3B* contributes to neuronal apoptosis after cerebral ischemia (Chuang et al., 2011) and cardiomyocyte death during MI (Nikolaou et al., 2019). Specifically, during cerebral ischemia, GSK-3β activation suppresses antioxidant responses and promotes inflammation (Rana and Singh, 2018), whereas its inhibition is neuroprotective (Rana and Singh, 2018; Chen et al., 2019). In MI, phosphorylation of GSK-3β at Ser9 increases the threshold for mitochondrial permeability transition pore opening, reducing cell death and myocardial injury (Sharma et al., 2021). These findings indicate that pharmacological modulation of GSK-3β represents a shared therapeutic strategy for mitigating cellular injury in both IS and MI.

To validate the predicted interaction with this key target, we performed molecular docking, which confirmed stable binding between all 10 core ingredients and GSK-3β (all binding energies < −5 kcal/mol), with Myricanone exhibiting the strongest affinity (−9 kcal/mol), providing strong structural evidence for direct targeting.

This computational prediction was experimentally validated by SPR, which confirmed the direct, concentration-dependent binding of Myricanone to GSK-3β, with a KD of 55.1 µM. This biophysical evidence provides direct experimental confirmation that Myricanone, a top-ranked core ingredient of BHD, can engage the shared key target GSK-3β with measurable affinity, thereby strongly supporting the network proximity and molecular docking predictions. Collectively, the convergence of computational and experimental evidence suggests that direct targeting of GSK-3β by ingredients such as Myricanone may represent a mechanism contributing to the therapeutic effects of BHD against IS and MI, warranting further functional investigation.

Despite these advances, the study has limitations. First, the use of public transcriptomic data entails limited sample sizes and platform heterogeneity, which may affect generalizability. Second, the use of a static PPI network fails to capture tissue- or cell-type-specific interactions; consequently, our analysis may overlook interactions specific to the brain (for IS) or heart (for MI), or those occurring only during acute ischemia. Third, although SPR confirmed direct binding between Myricanone and GSK-3β, the functional consequences, for example, effects on kinase activity and downstream signaling, have not been investigated in cellular or animal models of ischemic injury, nor have pharmacokinetic studies been performed. Fourth, candidate target identification relied primarily on transcriptomic data without independent validation cohorts or protein-level verification. Fifth, our ingredient analysis was limited to 842 BHD components with available target information; other constituents could not be assessed, and the potential synergistic effects among the nine remaining core ingredients remain unexplored.

## 5 Conclusion

In summary, this study reveals novel insights into the shared molecular mechanisms underlying IS and MI, identifies the “Lipid and atherosclerosis” pathway as a central convergent mechanism, and systematically elucidates the multi-target pharmacological basis of BHD. These findings provide a rationale for repurposing BHD as a multi-target therapeutic strategy for ischemic cardio-cerebrovascular diseases and highlight a promising candidate ingredient for future drug development.

## Data Availability

The original datasets GSE122709(IS), GSE109048, GSE60993, GSE61144(MI), and GSE274598(RA) analyzed in this study are publicly available in the GEO repository (https://www.ncbi.nlm.nih.gov/geo/). Disease–related genes were obtained from the MalaCards database (https://www.malacards.org/). The ingredient and target information for Buyang Huanwu Decoction and Guizhi Shaoyao Zhimu Decoction were sourced from the TCMKD database (https://cbcb.cdutcm.edu.cn/TCMKD/index). The data supporting the findings of this study are available within the article and its Supplementary Material. The custom scripts used for network analysis and proximity calculation are available from the corresponding author upon reasonable request.
