# Supplementary material for: Network-based analysis identifies shared mechanisms between ischemic stroke and myocardial infarction and therapeutic ingredients of Buyang Huanwu Decoction
Source: Front Genet. 2026 Jun 17;17:1843679. doi: 10.3389/fgene.2026.1843679 (PMC13318255; doi:10.3389/fgene.2026.1843679)
Supplement: Supplementary file 2 [file DataSheet1.docx]

Supplementary Material


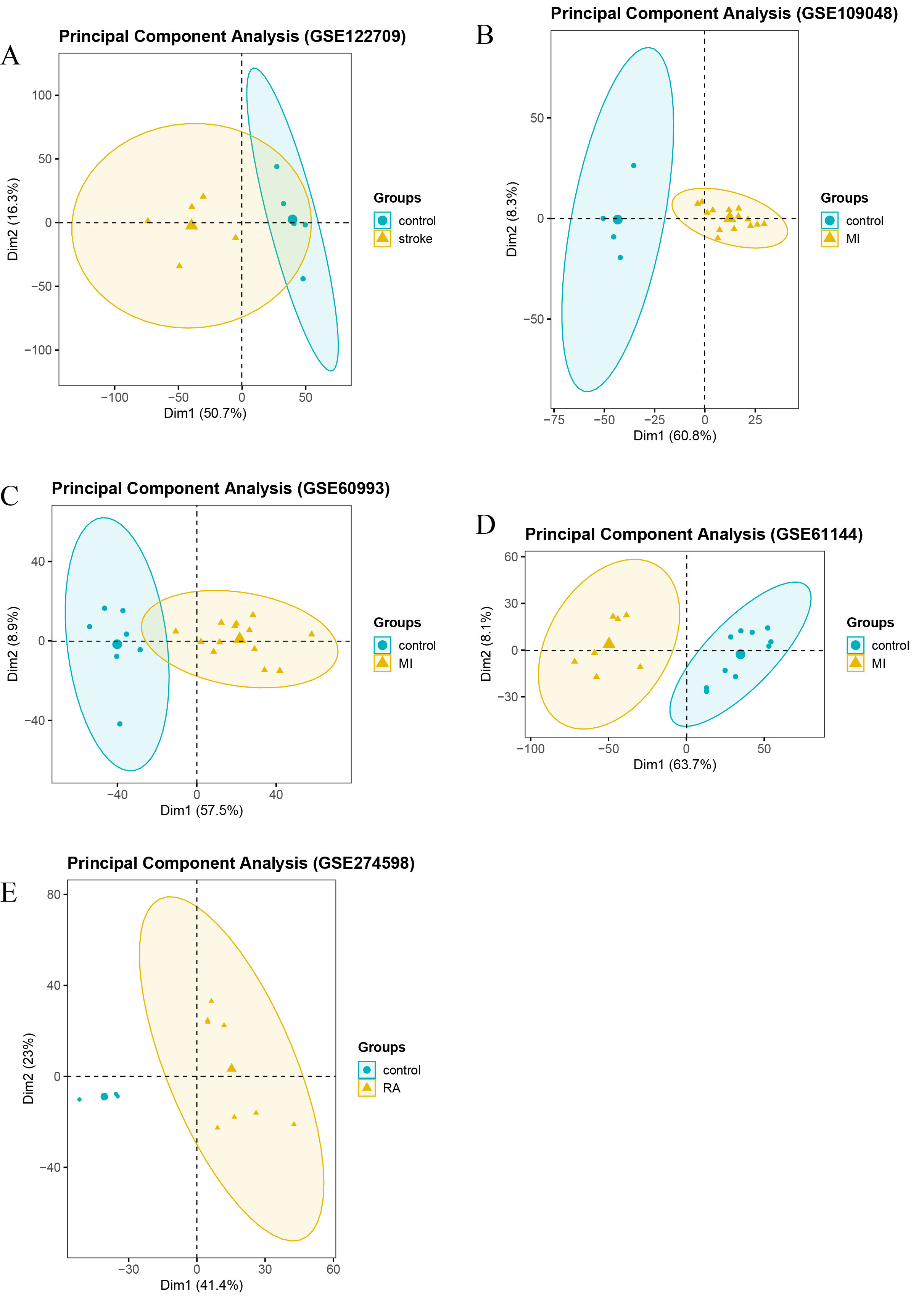


**Supplementary Figure S1.** PCA of transcriptomic data shows separation between disease and control groups. (A) IS dataset GSE122709, (B-D) MI datasets GSE109048, GSE60993, and GSE61144, respectively, and (E) RA dataset GSE274598.


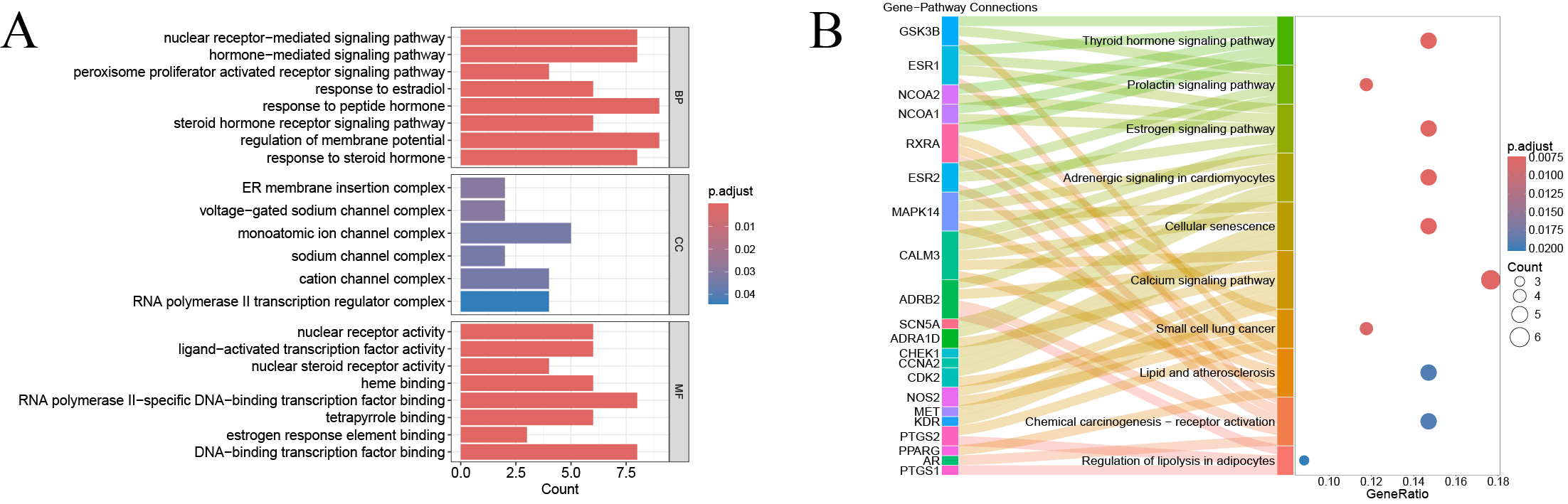


**Supplementary Figure S2.** Results of GO and KEGG enrichment analyses. (A) GO enrichment analysis of core ingredient targets, showing the top 8 terms in BP, CC, and MF ranked by adjusted *p*-value. (B) KEGG enrichment analysis of core ingredient targets, showing the top 10 pathways ranked by adjusted *p*-value.
